# A Bond-Level Sequence Framework for Molecular Representation Learning with Structural Constraints

**DOI:** 10.3390/molecules31111972

**Published:** 2026-06-05

**Authors:** Haoran Fan, Haoqiang Qi, Xin Huang, Dongyang Zhu, Na Wang, Ting Wang, Hongxun Hao

**Affiliations:** 1 National Engineering Research Center of Industrial Crystallization Technology, School of Chemical Engineering and Technology, Tianjin University, Tianjin 300072, China; fanhaoran@tju.edu.cn (H.F.); qhq_2469897703@tju.edu.cn (H.Q.); x_huang@tju.edu.cn (X.H.); zhudy@tju.edu.cn (D.Z.); 2 Engineering Research Center of Green Refining Process, Ministry of Education, Tianjin University, Tianjin 300072, China

**Keywords:** molecular property prediction, transformer, bond-level representation, self-supervised learning, structural degeneracy

## Abstract

Molecular property prediction is a fundamental task in drug discovery and materials design. While graph neural networks (GNNs) and SMILES-based Transformers have made significant strides, the former are often limited by local message-passing bottlenecks such as over-squashing, while the latter frequently lack explicit topological constraints and suffer from severe vocabulary imbalance. In this work, we revisit the granularity of molecular modeling and propose a representation learning framework built upon bond-level sequences. Our framework models molecules as sequences of directed bond tokens and introduces a structure-aware hybrid attention mechanism. By imposing hard topological constraints on a subset of attention heads to reinforce local connectivity while preserving global receptive fields in the remaining heads, the design is intended to separate short-range chemical bonding from long-range contextual dependencies. For pre-training, we implemented a multi-scale consistency learning paradigm, which utilizes an atom-centric group masking strategy to induce a hierarchical loss of local structural information and employs contrastive and triplet losses to ensure identity consistency across varying scales of structural degradation. Furthermore, by incorporating macro-scale physicochemical descriptors (e.g., LogP, TPSA) as global anchors, we examined how the inclusion of global attribute bias can provide weak physicochemical priors during pre-training, while its effect during downstream fine-tuning remains task-dependent. Experimental results demonstrate that our lightweight model, with approximately 3.5 million parameters, exhibits a dataset-dependent performance profile across MoleculeNet benchmarks and shows promising behavior on selected topology-sensitive tasks, particularly MUV. Ablation studies further analyze the contribution of bond-level connectivity, the stage-dependent dynamics of global attribute bias, structured masking, and pre-training configurations. Ultimately, this work provides an alternative representation design for molecular modeling, offering a parameter-efficient option for future molecular learning systems alongside traditional SMILES-based and graph-based formulations.

## 1. Introduction

Molecular property prediction plays a central role in drug discovery, materials design, and chemical informatics, enabling the efficient estimation of physicochemical properties and biological activities without extensive experimental measurements [1,2]. Generally, the core challenge of this task lies in molecular representation learning: how to encode molecular structures into expressive and transferable representations that faithfully capture structure–property relationships.

Molecules are naturally described as graphs, with atoms as nodes and chemical bonds as edges [3]. Consequently, graph neural networks (GNNs) have become a widely adopted paradigm for molecular representation learning, achieving strong performance across a wide range of benchmark datasets [4]. To mitigate the scarcity of labeled molecular data, recent studies have further introduced self-supervised pretraining strategies inspired by advances in natural language processing, such as masked language modeling [5]. In these approaches, atom types or local graph components are typically masked and reconstructed, encouraging models to learn general-purpose molecular representations from large-scale unlabeled data [6,7].

Despite their success, existing molecular pretraining frameworks still exhibit limitations that constrain their representational capacity. Firstly, most atom-level pretraining objectives rely on a small and highly imbalanced atom vocabulary, where a few atom types—such as carbon—dominate molecular datasets. This leads to limited supervision diversity and potentially biased learning signals [8]. Recent studies have further revealed that self-supervised pretraining does not consistently yield performance gains and can even induce negative transfer, a phenomenon largely attributed to the sparse or redundant information content of atom-level representations [9]. To enrich the supervisory signal, several works have introduced substructure- or fragment-level pretraining objectives, such as masking and predicting functional groups or molecular motifs [10,11]. However, these approaches often depend on predefined or dataset-specific structural units, raising concerns regarding their generality and robustness. Overall, existing pretraining research has predominantly focused on “how to design pretraining tasks,” while the question of “which representation unit is most suitable for pretraining”—and how it interacts with the underlying model architecture—remains underexplored.

Secondly, GNN-based architectures primarily depend on local message passing and are known to suffer from the over-squashing phenomenon, which limits their efficiency in capturing long-range dependencies such as extended conjugation effects or remote non-covalent interactions [12]. To address these atom-centric limitations, a line of research has shifted toward explicitly modeling chemical bonds and their directional properties. Architectures such as Hybrid Directional Graph Neural Networks (HDGNN) [13] incorporate directional information, while Directed Message Passing Neural Networks [14] use directed edges to prevent information backtracking. Line-graph-based GNNs further transform molecular graphs into line graphs to capture bond-level interactions and angular information [14]. These studies demonstrate the importance of bond- and edge-centric molecular modeling, indicating that the use of bonds as representation units is not itself a new concept. However, most existing bond-aware or line-graph-based methods are still implemented within graph message-passing or graph-convolutional frameworks, where capturing long-range dependencies generally requires multiple propagation layers [14,15]. This motivates the exploration of sequence-style formulations that can retain bond-level topological information while benefiting from the global receptive field of attention-based architectures.

Thirdly, sequence-based Transformer models built on SMILES representations provide global receptive fields and have achieved significant performance through large-scale pre-training, as demonstrated by MoLFormer-XL [16]. However, this success raises a fundamental question: does such dominance reflect an optimal representation choice, or does it arise primarily from the capacity of Transformers to compensate for syntactic imperfections through scale? Even with more robust descriptors like SELFIES [17], these approaches fundamentally treat molecules as one-dimensional strings, where learned correlations often reflect statistical co-occurrence patterns rather than the intrinsic chemical bond connectivity that governs molecular properties. Graph Transformers attempt to bridge this gap by injecting molecular topology through soft positional or distance biases [16,18]. Yet, these soft constraints tend to entangle local connectivity with long-range correlations, often struggling to prioritize the specific bond pathways that are essential for chemical reasoning. Consequently, there remains a need for a representation that can more faithfully interface with molecular topology while retaining the modeling flexibility of sequence-based architectures.

To address these limitations, in this work we explore a bond-level sequence formulation for Transformer-based molecular modeling. Bonds naturally encode relational information between atoms and exhibit substantially greater combinatorial diversity than atom types alone, which may help alleviate the issue of limited and imbalanced vocabularies. More fundamentally, chemical bonds serve as important pathways for chemical connectivity and electronic interactions. Unlike spatial proximity, which acts as a noisy proxy for interaction and may conflate geometric nearness with chemical connectivity, bond pathways define chemically meaningful routes of structural interaction. By modeling molecules as bond sequences, we can enforce clear structural constraints that guide models toward prioritizing these chemically meaningful paths without resorting to explicit graph-based message passing.

Motivated by these observations, herein we propose a bond-sequence representation framework for molecular property prediction, built entirely on attention mechanisms. Molecules are represented as sequences of bond-level tokens. Unlike methods that mainly rely on soft biases to encode structural information [18], we introduce a structure-aware hybrid attention mechanism that explicitly imposes hard topological constraints on a subset of attention heads to enforce local bond connectivity, while preserving unrestricted global attention in the remaining heads. This design aims to separate short-range chemical bonding from long-range contextual dependencies, enabling the model to jointly capture local structural priors and global molecular context within a unified Transformer architecture.

To learn transferable bond-level representations, we further introduce a masked bond token pretraining objective tailored to molecular structure. The pretrained encoder is subsequently fine-tuned for downstream molecular property prediction tasks. While the predictive performance varies across different datasets, the model demonstrates promising behavior on complex, topology-sensitive benchmarks such as MUV.

In summary, our study could contribute to the methodological integration of bond-centric topology into a sequence-based Transformer framework. We emphasize that the novelty of this work does not lie simply in using bonds as representation units, since bond- and edge-centric ideas have already been explored in D-MPNN, Chemprop-like models, line-graph GNNs, and graph Transformer frameworks. Instead, our main contribution lies in converting a line-graph-equivalent bond representation into a Transformer-compatible sequence framework and combining it with hard topological attention constraints and self-supervised pretraining. Specifically, our contributions are threefold:

1. We propose a deterministic conversion mechanism that transforms a 2D line-graph-equivalent bond representation into a 1D Transformer-compatible sequence, providing a sequence-based interface for bond-level molecular topology;
2. We design a structure-aware hybrid attention mechanism that utilizes a bond-to-bond adjacency matrix as a hard topological constraint. This aims to separate short-range chemical connectivity from unconstrained long-range global attention without relying on traditional absolute positional encodings;
3. We introduce an atom-centric structured masking pre-training strategy specifically tailored for bond-level sequences, aiming to reduce local information redundancy and encourage the learning of transferable topological representations.

## 2. Results and Discussion

### 2.1. Comparative Analysis and Performance Discussion

To evaluate our bond-sequence framework, we benchmarked its performance against three distinct categories of established baselines, ranging from classical supervised models to representative self-supervised architectures. Firstly, we selected representative supervised GNNs (GCN [19], GIN [20], and D-MPNN [15]) to establish the baseline performance achievable through pure topological modeling. Secondly, we contrasted our approach with self-supervised GNNs (MolCLR [21], GraphMAE [10], and GIN + ContextPred [5]) and advanced knowledge-guided Transformers (Mole-BERT [8], KPGT [22]) to assess the impact of bond-level tokenization. Notably, our architecture achieves these results with approximately 3.5 million parameters, representing a compact parameter scale compared with many larger molecular Transformer models.

The experimental results on molecular classification benchmarks (Table 1) reveal a dataset-dependent performance profile. On MUV, widely regarded as a challenging benchmark due to its design against structural artifacts, our model achieves an ROC-AUC of 77.64%. This is higher than the supervised baseline D-MPNN [15] (74.8%) and pre-training methods such as KPGT [22] (76.2%) and Mole-BERT [8] (77.3%). These results suggest that bond-level granularity may provide a useful inductive bias for capturing topology-sensitive structural patterns in selected tasks. Unlike atom-centric models, the bond-sequence representation naturally encodes bond connectivity patterns, which may help the Transformer represent chemically meaningful structural contexts.

On the large-scale HIV dataset, our model achieves 76.66%, showing performance comparable to established pre-training baselines like GraphMAE [10] (76.4%) and MolCLR [21] (77.0%), and exceeds standard supervised GCN [19]/GIN + ContextPred [5] models. While it trails slightly behind KPGT [22] (78.5%), it is important to contextualize this comparison. KPGT relies on more extensive external knowledge and molecular descriptors during its pre-training phase. In contrast, our model incorporates a lightweight set of four handcrafted physicochemical priors (TPSA, LogP, HBD, and HBA) calculated via RDKit. This highlights the smaller descriptor burden and lower parameter count of our framework, while acknowledging that it is not entirely free from external physicochemical knowledge.

Conversely, more conservative absolute scores are observed on certain datasets such as BBBP (62.65%) and ClinTox (71.77%). These results indicate that the proposed framework should not be interpreted as broadly superior across all benchmarks. While baseline models typically report optimal results derived from extensive, dataset-specific hyperparameter grid searches, our Bond-Transformer was evaluated under a unified, fixed hyperparameter protocol across all eight benchmarks. Furthermore, following the stage-dependent behavior observed in the global attribute ablation, we adopted the decoupled ptGA_ftNoGA setting to avoid directly imposing global descriptor bias during fine-tuning. Although this absence of task-specific tuning may limit absolute performance on some datasets, it provides a consistent assessment of the model under a unified evaluation setting.

Collectively, these findings suggest that the proposed bond-sequence framework offers a parameter-efficient and structurally aware alternative to existing molecular representation methods. By grounding the Transformer in a bond-level topological sequence and evaluating it under stringent unified protocols, our model combines local bond-connectivity constraints with global attention-based contextual modeling. This indicates that bond-centric sequence modeling may be particularly useful for selected complex or topology-sensitive molecular prediction tasks, while its performance remains dependent on the dataset and evaluation setting.

### 2.2. Ablation Study

#### 2.2.1. Topological Advantage of Bond-Level Representation

While chemical principles dictate that the electronic environment and conformational constraints of a molecule are intrinsically defined by the nature of its chemical bonds and their interaction networks, the potential advantage of bond-level representation can also be discussed from graph-theoretical principles. In a standard molecular graph G, the maximum degree of a carbon atom is naturally constrained by its valency to 4. However, by transforming the molecule into a bond-centric representation—conceptually equivalent to a line graph L(G)—the topological connectivity is altered and, in many cases, increased. The degree of a bond-level token e=(u,v) in L(G) is defined as

(1) deg(e)=deg(u)+deg(v)−2

For a typical C-C bond connecting two saturated carbons, this yields a degree of 6, creating a higher connectivity density that may facilitate information exchange within each attention layer.

To quantitatively support this observation, we conducted a statistical survey on a representative subset of the pre-training corpus. Our analysis reveals that while the traditional atom-level graphs exhibit an average node degree of 2.1071 and a graph density of 0.1144, the bond-level representation achieves a higher average degree of 2.7469 and a graph density of 0.1435. This corresponds to a 30.36% gain in connectivity density and a 25.43% increase in overall graph density, providing a richer topological context for the attention mechanism. Such enhanced density may help expose dense structural motifs, such as fused rings or highly branched chains, to the attention mechanism and partially alleviate the information bottlenecks associated with sparse atom-centric message passing.

#### 2.2.2. Impact of Global Attribute Bias

Beyond capturing local connectivity, our framework originally incorporates four global descriptors—Topological Polar Surface Area (TPSA), octanol-water partition coefficient (LogP), Hydrogen Bond Donors (HBD), and Hydrogen Bond Acceptors (HBA)—as macro-scale physicochemical anchors. Derived from Lipinski’s “Rule of Five,” ref. [23] these descriptors were intended to provide the model with a holistic profile of the molecular landscape. However, a pivotal observation is that the integration of these global biases significantly alters the model’s behavior depending on the training stage.

To thoroughly examine the role of these global attributes, we conducted a systematic cross-ablation study across the pre-training (pt) and fine-tuning (ft) stages (Table 2). Quantitatively, the results reveal a stage-dependent and task-dependent effect. During the pre-training phase, the inclusion of global attributes improves the masked bond modeling objective. As evidenced by the pre-training metrics, models incorporating global descriptors (ptGA) achieved a higher MBM accuracy (90.21%) compared to the “w/o Global” variant (83.28%), indicating that these descriptors can provide weak physicochemical priors that help guide representation learning during self-supervised pre-training.

However, the downstream results show that the direct use of the same global attributes during fine-tuning does not uniformly improve prediction performance. In several datasets, especially small or distribution-sensitive tasks, incorporating global attributes during fine-tuning can lead to lower ROC-AUC values compared with the decoupled setting. This observation suggests that global descriptors may introduce descriptor-induced negative transfer when their coarse-grained physicochemical bias is not well aligned with the task-specific structural patterns required by a downstream dataset.

Therefore, global attribute bias should not be interpreted as a universally beneficial regularizer. Instead, the ablation results support a stage-dependent usage pattern: global attributes can be useful during pre-training as weak macro-scale physicochemical priors, whereas their direct incorporation during fine-tuning should be treated cautiously and may need to be decoupled depending on the downstream task and data distribution.

#### 2.2.3. Synergy of Structured Masking and Multi-Scale Consistency Learning

Given the substantial computational overhead required to pre-train the Transformer encoder from scratch, we adopted a two-phase evaluation protocol. Phase 1 (Architectural Search): We conducted exploratory ablations (Table 3 and Table 4) using an initial parameter configuration fixed across variants. The primary objective of this phase was not to benchmark final fine-tuned performance, but to identify topological configurations that support numerical convergence and prevent representation collapse during pre-training. Phase 2 (Rigorous Evaluation): Once the selected stable architectural hyperparameters were locked (i.e., Structured Masking and r=0.85), we deployed the multi-seed protocol under a unified fine-tuning setting for the final baseline comparisons (Table 1) and the global attribute ablation (Table 2).

The design of the Atom-centric Structured Masking (SM) strategy is primarily motivated by the observation of inherent structural redundancy within bond-level sequences. Since adjacent bond tokens typically share common endpoint atoms, there is a potential risk that the model may exploit these local atom indices to perform trivial inference—a form of pattern matching that bypasses deep topological reasoning. By creating a “structural vacuum” through the simultaneous masking of all bonds connected to a central atom, this approach is intended to mitigate such local information leakage and steer the encoder toward capturing more robust molecular representations through non-redundant dependencies.

A critical finding from this Phase 1 architectural search (Table 3) is that Multi-scale Consistency Learning (MSCL) plays an important stabilizing role during pre-training. In configurations where MSCL was omitted (Groups 01 and 02), the model consistently suffered from representational collapse within the initial three epochs, with the training loss abruptly vanishing and the masked bond modeling accuracy dropping to zero. This phenomenon indicates that, due to the high-entropy nature of bond-level representations, the Transformer benefits from explicit semantic regularization to avoid converging toward non-informative, degenerate solutions. In this context, MSCL is not merely a performance-enhancing component but appears to be an important condition for stable optimization.

The synergy between SM and MSCL becomes most apparent when evaluating downstream task performance and predictive consistency. While the inclusion of MSCL alone enables stable convergence even with random masking, the integration of SM provides further performance gains in benchmarks sensitive to specific functional group arrangements, such as ClinTox (+2.96%) and BACE (+1.45%). Furthermore, the full model demonstrates improved predictive consistency; for instance, the standard deviation of predictions for the BACE task decreased from 1.04 to 0.29 when SM was employed alongside MSCL. These results suggest that the structural challenge posed by SM, when anchored by the semantic constraints of MSCL, may reduce the model’s reliance on local redundancies and encourage more transferable molecular representations.

#### 2.2.4. Quantifying Pre-Trained Knowledge Injection and Information Balancing

To evaluate the effect of our self-supervised learning phase, we compared the performance of the pre-trained encoder against a randomly initialized baseline (No Pre-train) and conducted a sensitivity analysis on the adjacency ratio r, which governs the integration of local structural focus and global context. As demonstrated in Table 4, the model initialized with random weights exhibits lower performance, particularly on large-scale and complex benchmarks. For instance, on MUV and Tox21, the pre-trained model (r=0.85) outperforms the non-pre-trained baseline by 11.15% and 7.41% respectively. This suggests that bond-level pre-training contributes useful transferable information beyond randomly initialized supervised learning under the same downstream setting.

A notable observation within our parametric sweep (r∈[0,1]) is the identification of a critical stability threshold. Specifically, when the adjacency ratio falls to or below 0.5 (r≤0.5), the pre-training process invariably suffers from representational collapse. This observation suggests that sufficient local structural bias is important for stabilizing the bond-level latent space during high-entropy reconstruction tasks. In the absence of robust local connectivity constraints, the model may fail to resolve the structural degeneracies induced by the masking procedure.

Among the stable configurations, our initially locked configuration of r=0.85 provides a favorable balance between local structural focus and global context in this sensitivity analysis. It outperforms the pure local structural representation (r=1.0) across almost all benchmarks, with notable gains in MUV (+5.42%) and HIV (+6.14%). This performance gap suggests that incorporating an appropriate proportion of global context during pre-training can complement local bond connectivity, although the utility of such global information remains stage-dependent and task-dependent. While r=1.0 remains numerically stable, its lower performance compared to r=0.85 suggests that excluding global context limits the model’s ability to contextualize local bond environments within the broader molecular architecture. Interestingly, on the smallest dataset (BBBP), performance exhibits limited sensitivity to pre-training and ratio adjustment, suggesting that the benefit of global-context balancing may be less pronounced in some small-sample settings.

#### 2.2.5. Exploration of Bond Orientation: Directional vs. Canonicalized Undirected Representation

A fundamental question in bond-level molecular modeling is whether the intrinsic ordering of atom pairs within a bond token—typically an artifact of graph traversal or computer storage—conveys meaningful chemical priors. In this study, we benchmarked our default directional representation (e.g., distinguishing between C=O and O=C) against a canonicalized undirected approach, which ensures a unique token for each chemical bond through predefined sorting protocols.

Empirical results (Table S1) demonstrate similar performance between the directional and canonicalized undirected strategies across all evaluated molecular property benchmarks. The performance divergence across the diverse range of datasets remains marginal, consistently falling within the range of standard deviation. This indicates that the Transformer architecture can obtain comparable predictive performance from these two bond-tokenization strategies under the current experimental setting.

From a computational perspective, the canonicalized undirected approach is more advantageous as it reduces the bond vocabulary size from 3804 to 2451 (a 35.6% reduction) without causing an obvious loss in predictive performance. This reduction in vocabulary complexity may reduce memory usage and simplify optimization during large-scale pre-training. Given that the increased task entropy of the directional representation does not translate into a tangible predictive gain, the undirected representation is a more compact and practical configuration for the present bond-level sequence framework.

### 2.3. Qualitative Representation and Attention Visualization

**Figure 1:**
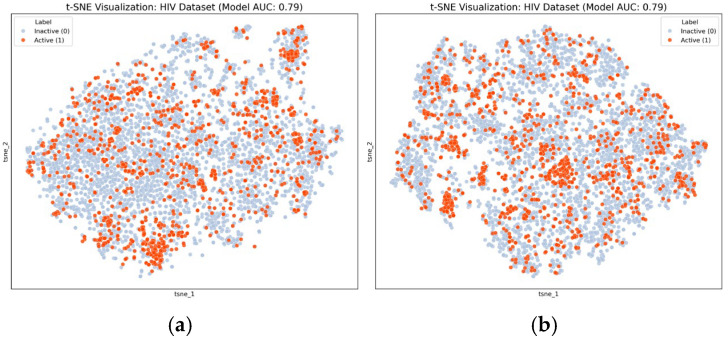
t-SNE visualization of molecular representations on the HIV dataset. (**a**) Embeddings extracted from the pre-trained model without fine-tuning. The mixture of active (orange) and inactive (blue) samples suggests that the pre-trained representations alone do not clearly separate HIV activity labels; (**b**) Embeddings extracted from the fine-tuned model. The emergence of localized active-sample clusters indicates that fine-tuning reshapes the latent space toward better task-related separation, although the visualization should be interpreted qualitatively.

To qualitatively examine the representations learned by the model, we performed a multi-scale visualization analysis, ranging from global latent space distribution to local attention distribution. Firstly, to inspect whether fine-tuning changes the representation space for HIV activity prediction, we visualized the latent features using t-SNE. Figure 1 compares the embeddings from the pre-trained and fine-tuned stages. As shown in Figure 1a, the pre-trained features of active (orange) and inactive (blue) molecules are highly entangled, indicating that generic structural features are insufficient for this task. In contrast, Figure 1b reveals a clear change in the latent distribution after fine-tuning. The emergence of dense, localized clusters of active molecules suggests that the model learns task-relevant discriminative patterns after fine-tuning. It is important to note that the remaining dispersion of some active molecules is chemically consistent with the intrinsic nature of the HIV dataset, which encompasses inhibitors targeting diverse mechanisms (e.g., reverse transcriptase vs. protease inhibitors) and possessing distinct scaffolds. Furthermore, the presence of “activity cliffs”—where minor structural changes drastically alter bioactivity—contributes to the complexity of the decision boundary. Despite these challenges, the clear transition from Figure 1a,b provides qualitative evidence that fine-tuning reshapes the representation space toward task-relevant separation.

To further explore whether the attention distribution shows qualitative correspondence with chemically relevant regions, we visualized the attention weights from the final Transformer layer for four representative active molecules. This analysis serves as a qualitative visualization: it first examines the attention pattern of Zidovudine (AZT) from the dense clusters in Figure 1b, and subsequently visualizes representative external clinical drugs (EFV, Raltegravir, and Saquinavir) that are not present in the original dataset.

**Figure 2:**
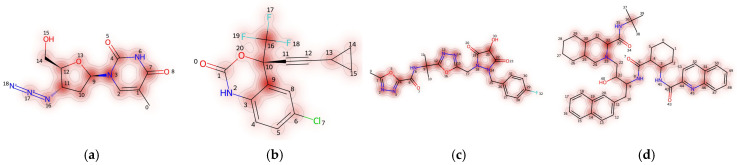
Qualitative attention visualization of the Bond-level Transformer. Heatmaps represent normalized attention weights from the final Transformer layer, where warmer colors indicate higher attention values. (**a**) AZT: higher attention is observed around the azido-containing region and the 5-methyl-2,4-dioxopyrimidine moiety. (**b**–**d**) Qualitative visualization on representative external drugs; (**b**) EFV shows relatively high attention around the electron-deficient aromatic region associated with the $-CF_{3}$ group; (**c**) Raltegravir shows higher attention around the chelation-related scaffold associated with $Mg^{2+}$ coordination; (**d**) Saquinavir shows higher attention near the transition-state-mimetic region. The numbers in the molecular diagrams indicate the atom indices.

As illustrated in Figure 2, the highlighted regions demonstrate a qualitative correspondence with reported pharmacophore-related regions. The analysis begins with AZT, a prototypical Nucleoside Reverse Transcriptase Inhibitor (NRTI). Relatively high attention weights are observed around the azido group ($-N_{3}$) moiety, while also assigning weight to the thymine (5-methyl-2,4-dioxopyrimidine) ring. This dual-focus pattern shows qualitative correspondence with the thymidine-mimicking structure of AZT, including the azido-containing region and the nucleobase moiety involved in enzyme recognition.

Moving to the external inhibitors, we observe a qualitative correspondence between the model’s attention weights and known structural motifs. In the case of Efavirenz (EFV), the attention assigns relatively high weights to the strongly electron-withdrawing trifluoromethyl group (-CF_3_) and the adjacent phenyl ring. This attention pattern shows qualitative correspondence with the electron-deficient aromatic region associated with known SAR features of EFV. This pattern of highlighting broader structural scaffolds is also observed in Raltegravir, where attention is distributed across the central hydroxypyrimidinone ring. This region corresponds to the chelation-related scaffold reported to participate in $Mg^{2+}$ coordination. Finally, for the complex peptidomimetic Saquinavir, the model assigns lower attention to the bulky peripheral hydrophobic naphthyl groups and assigns relatively higher attention to secondary hydroxyl group (−OH) and its adjacent nitrogen atom. This specific region shows qualitative correspondence with the transition-state-mimetic region involved in HIV protease inhibition.

Collectively, these qualitative observations suggest that the Bond-level Transformer assigns relatively higher attention to chemically plausible regions, such as pharmacophore-related motifs and functional groups. However, it is important to emphasize that these attention distributions serve solely as qualitative observations of pattern correlations rather than faithful mechanistic explanations. While the high-attention regions visually align with known pharmacological features, we cannot claim that the model has learned “electronic intuition” or moved “beyond simple pattern matching” based solely on attention weights. True chemical interpretability would require more rigorous attribution analyses in future work—such as integrated gradients, SHAP-like methods, input perturbations, or counterfactual molecular edits—to quantitatively compare the model’s decision patterns with reported Structure-Activity Relationship (SAR) trends.

## 3. Materials and Methods

### 3.1. Methodology

**Figure 3:**
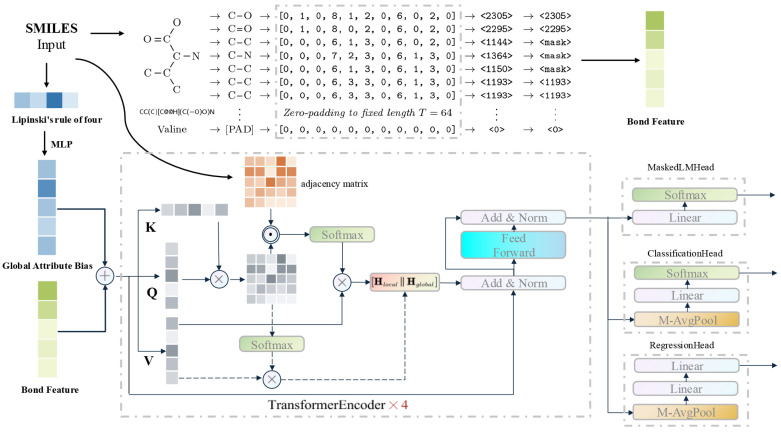
**Overall architecture of the bond-level molecular representation learning framework.** The framework converts SMILES strings into directed bond sequences featurized with intrinsic chemical attributes. Macro-scale context is incorporated via global physicochemical descriptors (e.g., TPSA, LogP) during pre-training as weak macro-scale priors. A structure-aware Transformer encoder utilizes a hybrid attention mechanism, where a bond-to-bond adjacency matrix A constrains specific heads to capture local topology ($H_{local}$) while others maintain global receptive fields ($H_{global}$). Final molecular representations are generated through masked mean pooling for joint optimization of Masked Bond Modeling (MBM) and downstream property prediction. Solid arrows represent primary data flows, while dashed arrows denote structural attention constraints.

In this study, we present a sequence-based framework for molecular representation learning that incorporates chemical structural information via attention constraints, without relying on explicit message-passing operations, as illustrated in Figure 3. The methodology consists of three components:

1. A bond-level preprocessing procedure that converts molecules into structured sequences;
2. A structure-aware self-attention architecture designed to separate local chemical constraints from global dependencies;
3. A specialized pre-training and fine-tuning strategy tailored for molecular property prediction.

#### 3.1.1. Preliminary

Given a molecule represented by a SMILES string, our goal is to construct an input representation that is compatible with sequence-based modeling while preserving essential chemical structural information.

Instead of operating on SMILES character sequences, we represent each molecule at the level of chemical bonds and treat it as a structured sequence of directed bond tokens (Figure 3, Top). To ensure reproducibility and determinism in sequence construction, we implement a canonicalization protocol. Using RDKit [24], each input SMILES string is first parsed into a canonical molecular graph, where atoms are assigned unique, deterministic canonical indices. We then iterate over all chemical bonds strictly following this canonical atom indexing order to construct the one-dimensional sequence. This ensures that alternative SMILES enumerations of the same molecule yield the same directed bond sequence under the canonicalization protocol.

Furthermore, unlike conventional NLP Transformers that rely on absolute positional encodings—rendering them highly sensitive to input token ordering—our architecture is less dependent on arbitrary token ordering. By discarding absolute positional encodings and relying on a structural bond-to-bond adjacency matrix (topological mask) within the self-attention mechanism, any alternative valid bond ordering can be handled by applying the corresponding permutation to both the token sequence and the adjacency matrix. This design reduces sensitivity to arbitrary sequence ordering and makes randomized-SMILES augmentation less central to this framework.

$N_{b}$ denotes the number of bonds in the molecule. For each bond i, we construct a fixed-dimensional feature vector $b_{i}\in R^{11}$ (Table 5). Following standard bond featurization practices adopted in bond-aware molecular models, ref. [25] these features encode intrinsic bond attributes (e.g., aromaticity, conjugation, and ring membership) as well as the atomic environments of the bond’s endpoints (e.g., atomic numbers, formal charges, hybridization states, total number of attached hydrogen atoms). Thus, a bond-level feature matrix could be obtained:

(2) $$B=[b_{1},b_{2},\ldots ,b_{N_{b}}{]}^{⊤}\in R^{N_{b}\times 11}$$

where each row corresponds to a single chemical bond.

For sequence modeling, we introduce a vocabulary-based tokenization scheme in which bond tokens are treated as directed entities. Specifically, for a chemical bond connecting atom u and atom v, the token corresponding to (u,bond,v) is distinct from (v,bond,u). This direction-sensitive formulation provides a controlled setting for examining whether directional tokenization introduces useful or redundant information. In particular, it enables an examination of whether the model learns to align reciprocal bond tokens—syntactically distinct yet chemically equivalent—within nearby regions of the embedding space, without enforcing explicit symmetry constraints.

Beyond individual bond features, structural relationships between bonds also play a critical role in molecular organization. Therefore, we define a bond-level connectivity matrix $A\in \{0,1{\}}^{N_{b}\times N_{b}}$, where $A_{ij}=1$ if bond *i* and bond *j* share at least one common atom. This bond-to-bond adjacency structure is equivalent to the line-graph representation of molecular graph, which specifies chemically valid pathways for information flow. Importantly, A is not used for recursive message passing or graph convolution. Instead, it serves as a hard structural constraint that guides interactions in the sequence-based attention mechanism.

#### 3.1.2. Model Architecture

Our model adopts a BERT-style Transformer architecture specifically tailored for bond-centric molecular sequences. Instead of relying solely on recursive message passing, the encoder combines global self-attention with explicit structural constraints, allowing interactions among bond tokens beyond immediate neighborhoods while preserving chemically meaningful local connectivity. This design complements the local aggregation paradigm of graph neural networks, which may be limited in capturing long-range electronic effects and extended conjugation patterns due to the over-squashing phenomenon [12].

The encoder follows a standard BERT-style, encoder-only design, refs. [26,27] consisting of a bond-token embedding layer, a stack of Transformer encoder layers, and task-specific output heads. Each molecule is represented as a sequence of implicit-hydrogen-bond tokens, which are mapped into continuous representations through a learnable embedding matrix before being processed by the self-attention layers.

A core challenge in molecular sequence modeling is the absence of an intrinsic linear order among chemical bonds. While positional encodings are commonly used in Transformers, imposing an artificial ordering is not naturally aligned with molecular topology and may introduce inductive biases unrelated to chemical structure. To incorporate molecule-level context without enforcing a predefined sequence order, we introduce a global attribute bias as an optional molecule-level conditioning term. For each molecule, a vector of physicochemical descriptors $a\in R^{d_{g}}$—typically based on macro-scale properties such as Lipinski’s rules (as illustrated in Figure 3, Left)—is projected into the hidden space through a learnable mapping ϕ(⋅):

(3) $$g=\phi (a)\in R^{d}$$

The resulting vector g is added as a shared bias to the initial representation of every bond token, providing molecule-level physicochemical conditioning that complements local structural information while reducing the need for artificial absolute positional encodings.

Structural information is incorporated into the self-attention mechanism through a hybrid attention design (Figure 3, Center). This mechanism separates representation learning by partitioning the total H attention heads into two functional groups: topology-constrained heads that aggregate local connectivity to form the local representation $H_{local}$, and unconstrained heads that capture long-range dependencies to form the global representation $H_{global}$. Specifically, let $n_{adj}=\left\lfloor H\cdot r\right\rfloor$ denote the number of topology-constrained heads. For the h-th attention head, the attention weight between bond tokens i and j is computed as

(4) $$\alpha_{ij}^{\left(h\right)}=soft\underset{j}{max}\left(\frac{q_{i}^{\left(h\right)}(k_{j}^{\left(h\right)}{)}^{⊤}}{\sqrt{d_{k}}}+M_{ij}^{\left(h\right)}\right)$$

where the structural mask $M_{ij}^{\left(h\right)}$ is defined as

(5) $$M_{ij}^{\left(h\right)}=\{\begin{array}{cc} 0, & h<n_{adj}\;and\;A_{ij}=1,\\ -\infty , & h<n_{adj}\;and\;A_{ij}=0,\\ 0, & h\geq n_{adj}. \end{array}$$

Herein, A denotes the bond-to-bond adjacency matrix derived from the molecular graph, equivalent to a line-graph representation. For topology-constrained heads, attention is restricted to chemically adjacent bonds, introducing a structural inductive bias analogous to local message passing, while the remaining heads are left unconstrained to capture longer-range dependencies and global context.

#### 3.1.3. Pre-Training and Fine-Tuning Strategy

**Figure 4:**
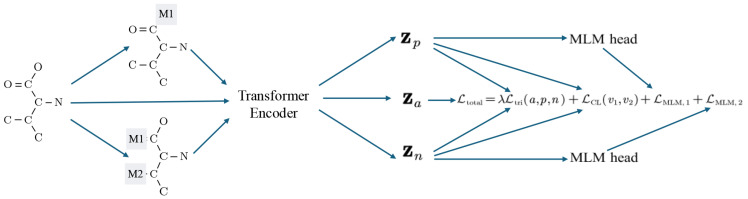
**Schematic of the multi-scale consistency pre-training strategy.** The framework generates hierarchical masked views (p and n) from an unmasked anchor molecule (a). A shared Transformer encoder maps these views into latent representations ($Z_{a},Z_{p},Z_{n}$), which are then optimized via a combination of Masked Bond Modeling and triplet consistency losses.

The model is first trained in a self-supervised manner on large-scale unlabeled molecular data and subsequently adapted to downstream molecular property prediction tasks. As illustrated in Figure 4, we implement a multi-scale consistency learning strategy that integrates local structural reconstruction with global semantic alignment [27]. Instead of vanilla masked modeling, we adopt an atom-centric structured group masking strategy to reduce the likelihood of trivial inference arising from local redundancy. For each molecular bond sequence, central atoms are randomly selected and all incident bonds are masked to create a local “information vacuum,” encouraging the model to reconstruct functional motifs (e.g., aromatic rings or conjugated systems) by leveraging broader structural context and reducing the structural ambiguities inherent in implicit-hydrogen bond sequences. To introduce hierarchical structural perturbations, the encoder processes three distinct views for each molecule (Figure 4): an unmasked anchor (a), a lightly perturbed view (p) with a 15% mask ratio, and a heavily perturbed view (n) with a 30% mask ratio. These views are mapped to latent vectors $Z_{a}$, $Z_{p}$, and $Z_{n}$ respectively through the shared Transformer encoder.

The pre-training objective minimizes a composite loss comprising a masked bond modeling term and semantic consistency constraints:

(6) $$L_{total}=(L_{mask1}+L_{mask2})+L_{CL}+\lambda L_{tri}$$

where the triplet loss $L_{tri}$ enforces a monotonic fidelity ranking in the latent space, encouraging the distance $d(Z_{a},Z_{p})$ to be smaller than $d(Z_{a},Z_{n})+\alpha$.

Following pre-training, the encoder is fine-tuned end-to-end for downstream molecular property prediction tasks. As depicted in the “Task Heads” section of Figure 3, a fixed-dimensional molecular representation is obtained by performing masked mean pooling (M-AvgPool) over the bond token embeddings, which is then fed into task-specific feed-forward layers for classification or regression.

For complete algorithmic details regarding sequence canonicalization, structured masking, and the topological forward pass, please refer to Algorithms S1–S3 in the Supplementary Materials.

### 3.2. Materials

#### 3.2.1. Evaluation Benchmarks

To evaluate the proposed bond-sequence modeling framework, we conducted experiments on a series of molecular property prediction benchmarks curated under the Open Graph Benchmark (OGB) framework. The selected datasets comprise eight widely used binary and multi-task prediction tasks: BACE, BBBP, ClinTox, HIV, MUV, SIDER, Tox21, and ToxCast. These benchmarks span diverse physiological and toxicological properties, ranging from blood–brain barrier penetration to large-scale stress response assays. By evaluating on these datasets, we provide an evaluation of the model across tasks with different biological endpoints and chemical complexity levels.

#### 3.2.2. Implementation Details

Experimental evaluations were conducted in accordance with the official data splitting and evaluation protocols established by the Open Graph Benchmark (OGB). For classification tasks, we adopted the official Bemis–Murcko scaffold-based splits to evaluate model generalization across chemically distinct molecular spaces, while regression tasks followed the standardized random splits. To ensure consistency with previous reports, all baseline results were directly cited from their original publications or standard leaderboards, which followed the corresponding benchmark splitting protocols. Furthermore, the benchmark results for our proposed model are reported as the average and standard deviation over three independent random seeds (i.e., 2026, 2027, and 2028). All benchmarks were performed under a unified two-dimensional molecular learning setting, where molecules were represented solely by their 2D topological structures without incorporating three-dimensional geometric coordinates.

Molecules were represented as directed bond-level token sequences and processed through a 4-layer structure-aware Transformer encoder comprising approximately 3.5 M trainable parameters. The model underwent self-supervised pre-training on one million molecules from the PubChem database, employing a multi-task objective that integrates Masked Bond Modeling (with dynamic 15% and 30% masking ratios) and a triplet contrastive loss. To provide macro-scale chemical context, four global physicochemical descriptors—TPSA, LogP, HBD, and HBA—were incorporated as a shared physicochemical bias during pre-training. For downstream evaluation, we adopted the decoupled ptGA_ftNoGA setting, in which the pre-trained encoder benefits from global descriptors during pre-training, whereas these descriptors are not directly imposed during fine-tuning. Detailed architectural and training hyperparameters, including learning rate schedules and optimization configurations, are provided in the Supporting Information and are summarized in Table S2, together with the exact random seeds and other optimization settings. Implementation specifics regarding the bond featurization protocol, software environment, and hardware specifications are also documented therein.

## 4. Conclusions

In this work, we explore an alternative perspective on molecular representation learning by revisiting the choice of representation granularity for Transformer-based models. Our study examines whether a bond-level representation can offer a structurally grounded and potentially scalable interface between molecular topology and attention-based sequence modeling. By representing molecules as sequences of directed bond tokens and implementing explicit topological constraints, we observe that Transformers can incorporate bond-level topological information without relying solely on traditional graph message passing.

One of the primary challenges addressed in this framework is the structural degeneracy encountered during high-entropy molecular pre-training. Our findings suggest that integrating atom-centric group masking with multi-scale consistency learning can help stabilize the pre-training process and improve representation consistency. In addition, the global attribute ablation indicates that macro-scale physicochemical descriptors can serve as weak priors during pre-training, whereas their direct use during fine-tuning should be treated as stage-dependent and task-dependent. Overall, the proposed approach is not intended to replace large-scale SMILES-based or graph-based methods, but to provide a parameter-efficient alternative representation design that is compatible with self-supervised objectives.

More broadly, this work is intended as a complementary viewpoint rather than a replacement for existing molecular modeling approaches. As Transformer architectures continue to dominate general-purpose representation learning, understanding how input units shape inductive bias remains a significant question beyond immediate benchmark performance. Looking ahead, the bond-centric granularity of our model provides a natural foundation for incorporating 3D geometric information. By extending the current binary topological mask to encode continuous physical descriptors such as bond angles (1st-order) and dihedral angles (2nd-order), this framework may provide a possible direction for connecting discrete bond-level topology with continuous conformational information. We hope that bond-level sequence modeling provides a useful reference point for future studies seeking to bridge discrete chemical structure with scalable sequence modeling frameworks, while further validation on broader datasets and more rigorous interpretability analyses remain important future work.

## Figures and Tables

**Table 1:** Comparative performance on molecular property prediction benchmarks across eight datasets. Performance is measured by ROC-AUC, with standard deviations provided in parentheses, using the official OGB scaffold splitting. The best and second-best results are **highlighted** in bold and underlined, respectively. “Supervised” denotes models trained from scratch without pre-training, and “-“ indicates results that are not reported or applicable.

Method	BBBP	Tox21	ToxCast	ClinTox	MUV	HIV	BACE	SIDER
GCN [19]	65.4 (1.2)	74.5 (1.1)	63.3 (0.9)	58.0 (4.4)	73.2 (1.4)	75.7 (1.1)	72.5 (2.1)	59.5 (1.1)
GIN [20]	65.8 (4.5)	74.0 (1.5)	63.4 (1.3)	58.0 (7.6)	71.8 (2.5)	75.3 (1.9)	70.1 (5.4)	57.3 (1.6)
D-MPNN [15]	71.0 (0.4)	74.5 (0.1)	67.9 (0.4)	**90.5 (1.5)**	74.8 (1.4)	78.1 (0.1)	**81.5 (0.6)**	63.2 (0.8)
GIN + ContextPred [5]	71.2 (0.9)	**78.3 (1.3)**	65.7 (1.3)	80.6 (1.4)	76.9 (1.6)	77.3 (1.0)	79.6 (1.2)	60.9 (0.6)
MolCLR [21]	72.0 (0.4)	75.0 (0.3)	64.8 (0.4)	89.0 (1.6)	73.0 (0.4)	77.0 (1.2)	71.5 (1.1)	60.0 (1.3)
GraphMAE [10]	72.0 (0.6)	75.1 (0.2)	63.0 (0.4)	80.5 (2.4)	75.7 (0.5)	76.4 (1.1)	81.3 (1.4)	58.5 (1.2)
Mole-BERT [8]	70.5 (1.5)	75.2 (0.2)	63.9 (0.6)	79.9 (1.9)	77.3 (0.4)	77.2 (0.7)	81.3 (1.6)	61.0 (0.6)
KPGT [22]	**72.8 (0.2)**	76.1 (0.1)	**66.5 (0.1)**	-	76.2 (0.5)	**78.5 (0.3)**	78.8 (0.7)	**64.3 (0.5)**
Bond-Transformer	62.65 (1.00)	70.97 (0.22)	61.46 (0.27)	71.77 (4.63)	**77.64 (0.51)**	76.66 (0.18)	72.94 (3.30)	56.64 (0.40)

**Table 2:** Ablation study of global attribute (GA) bias across pre-training(pt) and fine-tuning (ft) stages. Pre-training quality is reported as MBM Accuracy (%). Downstream performance across eight molecular property benchmarks is measured by ROC-AUC, with standard deviations reported in parentheses. To eliminate seed-selection bias, downstream results are averaged over three fixed random seeds (2026, 2027, 2028). The results suggest a stage-dependent effect of global attributes: they can provide weak physicochemical priors during pre-training, whereas their direct use during fine-tuning may introduce task-dependent negative transfer on certain downstream datasets.

Method	Pre-Training	BBBP	Tox21	ToxCast	ClinTox	MUV	HIV	BACE	SIDER
ptNoGA_ftNoGA	83.28	63.13 (0.91)	70.32 (0.18)	59.75 (0.22)	52.91 (10.93)	72.72 (0.52)	76.71 (0.88)	67.29 (1.15)	56.27 (1.09)
ptNoGA_ftGA	83.28	65.56 (0.64)	72.82 (0.26)	61.91 (0.20)	67.65 (3.38)	75.36 (0.55)	75.52 (1.50)	61.76 (1.80)	56.64 (1.38)
ptGA_ftNoGA	90.21	62.65 (1.00)	70.97 (0.22)	61.46 (0.27)	71.77 (4.63)	77.64 (0.51)	76.66 (0.18)	72.94 (3.30)	56.64 (0.40)
ptGA_ftGA	90.21	63.29 (0.26)	70.26 (0.40)	60.56 (0.35)	59.20 (5.78)	77.29 (0.78)	74.72 (1.32)	64.23 (2.05)	50.33 (0.47)

**Table 3:** Ablation study of masking strategies and multi-scale consistency learning (MSCL) across eight molecular property benchmarks. Performance is measured by ROC-AUC, with standard deviations reported in parentheses. “Collapsed” indicates that the model failed to converge during pre-training *.

Masking	MSCL	BBBP	Tox21	ToxCast	ClinTox	MUV	HIV	BACE	SIDER
Random	w/o	Collapsed							
Structured	w/o	Collapsed							
Random	w/	66.22 (0.41)	74.82 (0.45)	64.31 (0.37)	72.70 (0.75)	78.68 (0.06)	76.62 (0.58)	68.67 (1.04)	57.40 (0.94)
Structured	w/	66.53 (0.31)	74.41 (0.10)	64.60 (0.19)	75.66 (1.47)	79.30 (0.90)	77.13 (0.72)	70.12 (0.29)	57.97 (0.85)

* Note: This ablation was performed during Phase 1 (Architectural Search) to identify configurations that prevent pre-training collapse. The optimal stable architecture was then frozen for the final multi-seed benchmarks presented in Table 1 and Table 2. “w” means “with”; “w/o” means “without”.

**Table 4:** Sensitivity analysis of the adjacency ratio r and the impact of pre-training. Performance is measured by ROC-AUC, with standard deviations reported in parentheses. “No Pre-train” refers to the model initialized with random weights. “Collapsed” indicates that the model failed to achieve numerical convergence during the pre-training phase due to representational instability. The ratio r represents the structural focus weight, where *r* = 1.0 denotes a pure local structural focus *.

Masking	BBBP	Tox21	ToxCast	ClinTox	MUV	HIV	BACE	SIDER
No Pre-train	66.60 (0.10)	67.00 (2.05)	54.56 (0.22)	51.32 (1.27)	68.15 (0.29)	71.09 (2.30)	68.06 (0.41)	49.16 (1.10)
*r* = 0				Collapsed				
*r* = 0.25				Collapsed				
*r* = 0.50				Collapsed				
*r* = 0.75	65.48 (1.07)	72.64 (0.17)	60.87 (0.32)	72.12 (3.04)	76.41 (1.48)	74.93 (1.89)	64.60 (3.54)	58.69 (0.47)
***r* = 0.85** **(Initial Config)**	**66.53 (0.31)**	**74.41 (0.10)**	**64.60 (0.19)**	**75.66 (1.47)**	**79.30 (0.90)**	**77.13 (0.72)**	**70.12 (0.29)**	**57.97 (0.85)**
*r* = 1.0	65.83 (0.11)	73.52 (0.33)	62.40 (0.10)	73.55 (0.48)	73.88 (1.31)	70.99 (1.19)	65.47 (3.57)	55.25 (0.40)

* Note: Similar to Table 3, this sensitivity analysis was conducted during Phase 1 to determine pre-training structural stability. Final evaluations of the locked architecture are presented in Table 1 and Table 2.

**Table 5:** **Definition of the 11-dimensional bond feature vector.** The vector concatenates intrinsic bond attributes with the atomic features of the two connected atoms.

Domain	Feature	Dim.	Description
Bond	Ring Status	1	Whether the bond is part of a ring
	Conjugation	1	Whether the bond is conjugated
	Aromaticity	1	Whether the bond is aromatic
Atom (Head/Tail)	Atomic Number	2 *	Atomic numbers of connected atoms
	Chirality/Hybridization	2	Hybridization state/Chirality tag
	Formal Charge	2	Electrical charge of the atom
	Num. Hydrogens	2	Count of attached hydrogen atoms
Total		11	

* Note: Dimensions of 2 for atom-level features denote the concatenated values of the two respective bond endpoints.

## Data Availability

The source code, environment specifications, and a self-contained demonstration dataset supporting the findings of this study are publicly available in the official repository to ensure reproducibility and open access: [https://github.com/Haoran-Fan/bondformer] (accessed on 2 June 2026).

## Supplementary Materials

The following supporting information can be downloaded at: https://www.mdpi.com/article/10.3390/molecules31111972/s1, Figure S1: Stability and convergence analysis across architectural configurations; Figure S2: Statistical distribution of molecular sizes and elemental compositions in the pre-training dataset; Table S1: Impact of bond orientation on downstream performance and vocabulary complexity; Table S2: Comprehensive hyperparameters used in the pre-training and downstream fine-tuning stages; Table S3: Theoretical computational complexity and parameter estimation comparison across different molecular representation modalities; Algorithm S1: Vectorized Tokenization Mechanism of Molecular Multidimensional Physical Attributes; Algorithm S2: Atom-Centric Structured Masking and Multi-Scale View Construction; Algorithm S3: Structure-Aware Bond-Level Transformer Encoder Forward Pass.

## Abbreviations

The following abbreviations are used in this manuscript:

AZT	Zidovudine (Azidothymidine)
BACE	Beta-site Amyloid Precursor Protein Cleaving Enzyme
BBBP	Blood–Brain Barrier Penetration
BERT	Bidirectional Encoder Representations from Transformers
ClinTox	Clinical Toxicity
D-MPNN	Directed Message Passing Neural Network
EFV	Efavirenz
GCN	Graph Convolutional Network
GIN	Graph Isomorphism Network
GNNs	Graph Neural Networks
HBA	Hydrogen Bond Acceptors
HBD	Hydrogen Bond Donors
HDGNN	Hybrid Directional Graph Neural Networks
HIV	Human Immunodeficiency Virus
KPGT	Knowledge-Guided Pre-training of Graph Transformer
LogP	Octanol-water partition coefficient
MAE	Mean Absolute Error
MBM	Masked Bond Modeling
MolCLR	Molecular Contrastive Learning of Representations
MSCL	Multi-scale Consistency Learning
MUV	Maximum Unbiased Validation (an exceptionally challenging virtual screening benchmark designed to eliminate structural target bias through refined spatial clustering and extreme data sparseness).
NRTI	Nucleoside Reverse Transcriptase Inhibitor
RDKit	Open-source Cheminformatics Software
RMSE	Root Mean Square Error
ROC-AUC	Area Under the Curve of the Receiver Operating Characteristic
SELFIES	Self-Referencing Embedded Strings
SIDER	Side Effect Resource
SMILES	Simplified Molecular Input Line Entry System
Tox21	Toxicology in the 21st Century
ToxCast	Toxicity Forecaster
TPSA	Topological Polar Surface Area
