# Supplementary material for: A Bond-Level Sequence Framework for Molecular Representation Learning with Structural Constraints
Source: Molecules. 2026 Jun 5;31(11):1972. doi: 10.3390/molecules31111972 (PMC13257498; doi:10.3390/molecules31111972)
Supplement: Supplementary file 1 [file molecules-31-01972-s001.zip › molecules-4343444-supplementary.pdf]

# Supplementary Information

## A Bond-Level Sequence Framework for Molecular Representation Learning with Structural Constraints

Haoran Fan, Haoqiang Qi, Xin Huang , Dongyang Zhu, Na Wang, Ting Wang and Hongxun Hao

## Experimental and Implementation Details

### Hardware Configuration

All computational experiments, including large-scale self-supervised pre-training and downstream property prediction fine-tuning, were conducted on a high-performance Linux workstation. The hardware environment is equipped with an Intel(R) Core(TM) i9-14900K CPU (24 cores, 32 threads) and 128 GB of DDR5 RAM. To accelerate deep learning operations and handle intensive attention mechanism computations, we utilized an NVIDIA GeForce RTX 3090 GPU with 24 GB of GDDR6X video memory (VRAM). This configuration provided sufficient throughput and memory capacity for processing complex bond-level molecular sequences and large batch sizes.

### Software Environment

The framework was developed and executed on Ubuntu 24.04.4 LTS (Noble Numbat). The core modeling pipeline was implemented using Python 3.9.23 and PyTorch 2.7.1. For GPU-accelerated tensor operations, CUDA 11.8 was employed as the compute platform. Chemical informatics operations, including SMILES parsing, graph-to-sequence conversion, and bond-level attribute extraction, were performed using RDKit version 2025.03.5. All experiment management and dependency isolation were maintained via Conda virtual environments to ensure full technical reproducibility.

### Pre-training Implementation

The self-supervised pre-training phase was executed for 20 epochs with a total of 116,340 optimization steps. To ensure reproducibility across different environments, a fixed random seed of 2028 was applied to all data shuffling and weight initialization procedures. We employed the AdamW optimizer with a weight decay of 0.01. The learning rate followed a linear warmup strategy for the first 5,817 steps (approximately 5% of the total schedule) until reaching a peak value of $1\times10^{-5}$, after which it was decayed using a cosine annealing scheduler. To optimize computational efficiency and memory throughput on the RTX 3090 GPU, Automated Mixed Precision (AMP) was enabled throughout the pre-training process. Gradient clipping with a maximum norm of 5.0 was further utilized to maintain numerical stability during the high-entropy masked reconstruction task.

### Loss Configurations

The composite pre-training objective $\mathcal{L}_{\text{total}}$ was optimized with the consistency loss weight $\lambda_{cl}$ and triplet loss weight $\lambda_{tri}$ both set to 1.0. The contrastive temperature $\tau$ was maintained at 0.1. For the semantic fidelity ranking, the triplet margin $\alpha$ was empirically fixed at $1\times10^{-6}$.

**Fine-tuning Specifics.**

Following the OGB standard protocols, downstream fine-tuning was performed for 50 epochs. We implemented a differential learning rate strategy, where the pre-trained encoder's learning rate was restricted to 5% of the task-specific head's learning rate ($2\times10^{-5}$) to preserve learned chemical priors. An initial 5-epoch freeze period for the encoder was enforced to allow for the stable initialization of task-specific layers. For benchmarks with high label imbalance, a square-root-based positive weight (sqrt) was incorporated into the loss function to enhance model sensitivity toward rare molecular properties.

**Impact of bond orientation**

**Table S1. Impact of bond orientation on downstream performance and vocabulary complexity.** Performance is measured by ROC-AUC, with standard deviations reported in parentheses, across representative molecular benchmarks. Vocabulary size refers to the total number of unique bond tokens defined in the pre-training framework. The results demonstrate that the canonicalized undirected representation achieves a significant reduction in vocabulary size (35.6%) while maintaining performance parity across all evaluated tasks.

| Method | size | BBBP | Tox21 | ToxCast | ClinTox | MUV | HIV | BACE | SIDER |
|---|---|---|---|---|---|---|---|---|---|
| w/o Global | 2451 | 66.53(0.31) | 72.76(0.17) | 63.61(0.23) | 86.47(0.49) | 74.99(1.84) | 76.48(0.25) | 83.56(0.44) | 62.39(0.16) |
| Full | 3804 | 66.53(0.31) | 74.41(0.10) | 64.60(0.19) | 75.66(1.47) | 79.30(0.90) | 77.13(0.72) | 70.12(0.29) | 57.97(0.85) |

**Architectural Parameter Estimation and Computational Complexity Analysis**

To substantiate the lightweight nature of the proposed framework, a rigorous mathematical derivation of the model's parameter scale and theoretical computational complexity is provided. As systematically summarized in Table S3, the estimated parameter count of approximately 3.5M for the Bond-Transformer is directly derived from its compact architectural configuration: a vocabulary size $V \in \{2451,3804\}$ depending on specific configurations, $L_{layers} = 4$Transformer layers, a token embedding dimension $d_{model} = 256$ , and a feed-forward network hidden dimension $d_{ffn} = 1024$ . The exact and comprehensive hyperparameter protocol utilized across all experiments is detailed in Table S3. Excluding minor task-specific output projection heads, the total number of trainable backbone parameters is formally estimated as follows:

$$\text{Parameters} \approx (V \times d_{model}) + L_{layers} \times \left(4d_{model}^2 + 2d_{model}d_{ffn}\right) \approx 3.5 \times 10^6$$

Beyond parameter efficiency, this bond-level sequence design inherently addresses the computational bottlenecks found in text-based molecular modeling. Due to the syntax rules of molecular notation, the total number of actual chemical bonds ($K$) in any given molecule is significantly smaller than the character

length of its corresponding SMILES string ($L$), which is heavily padded with redundant structural characters such as branching brackets, ring-closure numbers, and explicit aromatic indicators, thus strictly satisfying $K \ll L$. Since the theoretical computational complexity of the Transformer's self-attention mechanism scales quadratically with respect to input length ($O(\text{Length}^2)$), our formulation compresses the operational FLOPs per block to approximately $1/4$ to $1/9$ of standard SMILES-based Transformers (as detailed in Table S2). This mathematical reduction explicitly justifies the extreme computational efficiency of our framework and demonstrates its viability for efficient training and inference on consumer-grade hardware.

**Table S2. Comprehensive hyperparameters used in the pre-training and downstream fine-tuning stages.**

| Hyperparameter | Pre-training Stage | Fine-tuning Stage |
|---|---|---|
| **Model Architecture** | | |
| Transformer layers | 4 | 4 (Shared backbone) |
| Token embedding dimension | 256 | 256 (Shared backbone) |
| Hidden dimension (FFN) | 1024 | 1024 (Shared backbone) |
| Number of attention heads | 32 | 32 (Shared backbone) |
| Adjacency ratio | 0.85 | 0.85 (Shared backbone) |
| Max sequence length | 64 bonds | 64 bonds |
| **Optimization & Training** | | |
| Optimizer | AdamW | AdamW |
| Base learning rate | $1\times10^{-5}$ | Head: $2\times10^{-5}$; Encoder: $1\times10^{-6}$ |
| Learning rate schedule | Linear warmup (2000 steps), then constant | Cosine annealing ($\eta_{\min}=10^{-6}$) |
| Weight decay | 0.01 | 0.01 |
| Gradient clipping | 5.0 | 5.0 |
| Batch size | 256 | 32 |
| Max epochs | 30 | 50 |
| Dropout rate | 0.1 | 0.2 |
| Early stopping patience | N/A | 10 epochs (based on validation AUC) |
| Fine-tuning protocol | N/A | 5 epochs with frozen encoder, then fully unfreeze |
| **Objective Functions** | | |
| Masking strategy | Atom-centric structured masking | N/A |
| Masking ratios | 15% (light view), 30% (heavy view) | N/A |
| Loss components | $\mathcal{L}_{\text{MLM}} + \lambda_{\text{cl}}\mathcal{L}_{\text{cl}} + \lambda_{\text{tri}}\mathcal{L}_{\text{tri}}$ | Multi-label BCEWithLogitsLoss[a] |
| Triplet margin | $1\times10^{-6}$ | N/A |
| Contrastive temperature | 0.1 | N/A |

[a] *The positive weight for the Binary Cross-Entropy loss is dynamically scaled using the square root of the negative-to-positive sample ratio to address severe class imbalance in downstream benchmarks.*

**Table S3. Theoretical computational complexity and parameter estimation comparison across different molecular representation modalities.**

| Method | Backbone Architecture | Pre-training Modality | Parameter Count (M) | Theoretical Computational Cost (FLOPs / Block) | Fine-tuning Footprint |
|---|---|---|---|---|---|
| KPGT [22] | LiGhT (Line Graph Transformer) | Dual-view Graph (Large) | ~42M to 80M | $O(N^2 \cdot D)$ (Large Line-Graph Matrix) | Heavy (Requires High VRAM) |
| Mole-BERT [8] | GNN + VQ-VAE Style | Atom-centric Graph | ~15M to 30M | $O(E \cdot D)$ (Iterative Message Passing) | Medium |
| MolFormer-XL* | Standard Transformer | SMILES Sequence (1D) | ~43M | $O(L^2 \cdot D)$ (Long SMILES Sequences,$L \approx 128$) | Heavy |
| Bond-Transformer (Ours) | Custom Transformer | Bond-level Sequence (1D) | 3.5M | $O(K^2 \cdot D)$ (Short Token Sequences,$K \ll L$) | Extremely Lightweight |

* MolFormer-XL is included as the representative SMILES-based foundation molecular Transformer requested by the reviewer, with its architectural statistics extracted from its originally published source. $N$, $E$, $L$, and $K$ denote the number of line-graph nodes, molecular graph edges, SMILES string characters, and our compressed bond-level tokens, respectively, while $D$ represents the hidden dimension.

**Algorithm S1:** Vectorized Tokenization Mechanism of Molecular Multidimensional Physical Attributes

**Input:** Molecular SMILES string $s$, maximum bond sequence length $L_{\max}$
**Initialize:** $\mathcal{G} \leftarrow \text{MolFromSmiles}(s)$ ▷ Parse into molecular graph topology
**Initialize:** $\mathcal{E}_b \leftarrow \text{GetDirectedBonds}(\mathcal{G})$ ▷ Extract directed bond set
**Initialize:** $\mathcal{V}_{vec} \leftarrow [], \mathcal{V}_{pos} \leftarrow []$
**for each** $e_i = (u, v) \in \mathcal{E}_b$ **do**
  $\mathbf{v}_i \in \mathbb{R}^{11} \leftarrow [f_{\text{atom}}(u), f_{\text{atom}}(v), f_{\text{bond\_type}}(e_i), \dots, f_{\text{hyb}}(v)]^\top$
  $\mathbf{p}_i \in \mathbb{N}^2 \leftarrow [\text{idx}(u), \text{idx}(v)]^\top$
  $\mathcal{V}_{vec} \leftarrow \mathcal{V}_{vec} \cup \{\mathbf{v}_i\}, \mathcal{V}_{pos} \leftarrow \mathcal{V}_{pos} \cup \{\mathbf{p}_i\}$
**end for**
$\mathcal{S} = (t_1, t_2, \dots, t_N) \leftarrow \text{VocabMap}(\mathcal{V}_{vec})$ ▷ Vocabulary ID mapping
$\mathbf{P} \in \mathbb{N}^{N \times 2} \leftarrow \text{Tensorize}(\mathcal{V}_{pos})$
**if** $N > L_{\max}$ **then**
  $\mathcal{S}, \mathbf{P} \leftarrow \mathcal{S}[: L_{\max}], \mathbf{P}[: L_{\max}]$ ▷ Truncate oversized sequence
**else**
  $\mathcal{S}, \mathbf{P} \leftarrow \text{Padding}(\mathcal{S}, \mathbf{P}, L_{\max})$ ▷ Pad to $L_{\max}$ with <PAD>
**end if**
**return** $\mathcal{S}, \mathbf{P}$ ▷ Token ID sequence $\mathcal{S} = (t_1, t_2, \dots, t_N)$
▷ Topological position matrix $\mathbf{P} \in \mathbb{N}^{N \times 2}$

**Algorithm S2:** Atom-Centric Structured Masking and Multi-Scale View Construction

**Input:** Molecular graph $\mathcal{G} = (\mathcal{V}, \mathcal{E})$, light/heavy mask ratio $\rho_p/\rho_n$
**Initialize:** $\mathcal{S} = (t_1, t_2, \dots, t_N) \leftarrow \phi(\mathcal{G})$ ▷ Call Algorithm S1
$a \leftarrow \mathcal{S}$ ▷ Original unmasked view
**for** $(view, \rho) \in \{(p, \rho_p), (n, \rho_n)\}$ **do**
  $view \leftarrow \mathcal{S}$
  $k \leftarrow \lceil N \cdot \rho \rceil$
  $M \leftarrow \emptyset, \mathcal{C} \leftarrow \emptyset$
  **while** $|M| < k$ **do**
    $v_c \sim \mathcal{V} \setminus \mathcal{C}$ ▷ Uniformly sample center atom
    $M \leftarrow M \cup \{e \in \mathcal{E} \mid v_c \in e\}$ ▷ Mask all adjacent bonds of $v_c$
    $\mathcal{C} \leftarrow \mathcal{C} \cup \{v_c\}$
  **end while**
  $view[M] \leftarrow$ [MASK]
**end for**
**return** Original view $a$, lightly perturbed view $p$, heavily perturbed view $n$

**Algorithm S3:** Structure-Aware Bond-Level Transformer Encoder Forward Pass

> 1. **Input:** Molecular graph $\mathcal{G} = (\mathcal{V}, \mathcal{E})$, global attribute vector $\mathbf{g}$, adjacency ratio $r$, number of attention heads $H$

> 2. **Initialize:** $\mathcal{S} = (t_1, t_2, \dots, t_N) \leftarrow \phi(\mathcal{G})$ ▷ Call Algorithm S1

> 3. $\mathbf{H}^{(0)} \leftarrow \text{Embed}(\mathcal{S}) \in \mathbb{R}^{N \times d}$ ▷ Initial feature mapping

> 4. $\gamma, \beta \leftarrow \phi_\gamma(\mathbf{g}), \phi_\beta(\mathbf{g})$

> 5. $\mathbf{H}^{(0)} \leftarrow \gamma \odot \mathbf{H}^{(0)} + \beta$ ▷ Global physical attribute modulation

> 6. $\mathbf{A} \in \{0,1\}^{N \times N} \leftarrow \text{GetAdjacency}(\mathcal{E})$ ▷ $\mathbf{A}_{ij} = 1$ $i$ndicates two bonds share an atom

> 7. Construct topological mask matrix **M:**

$$\mathbf{M}_{ij} = \begin{cases} 0, & \mathbf{A}_{ij} = 1 \\ -\infty, & \mathbf{A}_{ij} = 0 \end{cases}$$

> 8. $n_{adj} \leftarrow \lfloor H \cdot r \rfloor$ ▷ Calculate the number of local topological heads

> 9. **for** $h = 1$ **to** $H$ **do**

> 10. $\quad \mathbf{Q}_h, \mathbf{K}_h, \mathbf{V}_h \leftarrow \mathbf{H}^{(l)}\mathbf{W}_h^Q, \mathbf{H}^{(l)}\mathbf{W}_h^K, \mathbf{H}^{(l)}\mathbf{W}_h^V$

> 11. $\quad \mathbf{E}_h \leftarrow \frac{\mathbf{Q}_h \mathbf{K}_h^\top}{\sqrt{d_k}}$

> 12. $\quad$ **if** $h \le n_{adj}$ **then**

> 13. $\quad\quad \mathbf{E}_h \leftarrow \mathbf{E}_h + \mathbf{M}$ ▷ Inject local topological hard constraint

> 14. $\quad$ **end if**

> 15. $\quad \mathbf{A}_h \leftarrow \text{Softmax}(\mathbf{E}_h)$

> 16. $\quad \mathbf{head}_h \leftarrow \mathbf{A}_h \mathbf{V}_h$

> 17. **end for**

> 18. $\mathbf{H}_{attn} \leftarrow \text{Concat}(\mathbf{head}_1, \dots, \mathbf{head}_H)\mathbf{W}^O$

> 19. $\mathbf{H}^{(l+1)} \leftarrow \text{LayerNorm}(\mathbf{H}^{(l)} + \text{FFN}(\text{LayerNorm}(\mathbf{H}^{(l)} + \mathbf{H}_{attn})))$

> 20. **return** $\mathbf{H}^{(l+1)}$ ▷ Bond-level token representation matrix $\mathbf{H}^{(l+1)}$

## Supplementary Figures

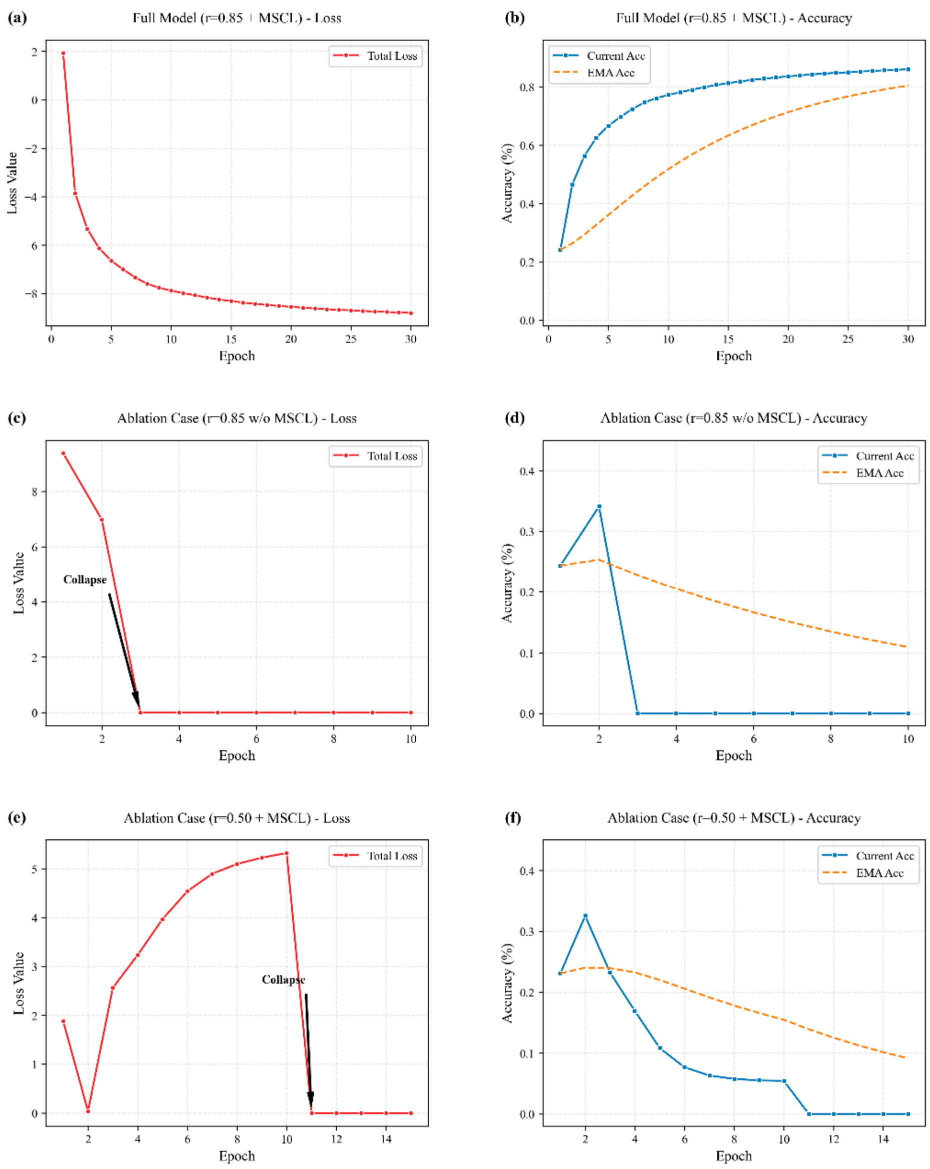


**Figure S1. Stability and convergence analysis across architectural configurations**. (a, b) Full Model (r=0.85 + MSCL): Demonstrates robust, stable convergence in total loss and reconstruction accuracy, serving as a benchmark for balanced structural and semantic constraints. (c, d) Ablation Case (r=0.85 w/o MSCL): Exhibits immediate representational collapse by the third epoch, with loss abruptly vanishing and accuracy dropping to zero. This underscores that structural constraints alone are insufficient for stable bond-level learning without semantic anchoring from Multi-scale Consistency Learning (MSCL). (e, f) Ablation Case (r=0.50 + MSCL): Reveals a delayed collapse at Epoch 11, signifying that while MSCL enhances stability, a proper adjacency ratio remains critical; over-reliance on global dependencies (r=0.50) introduces high-entropy noise that eventually destabilizes the latent space.

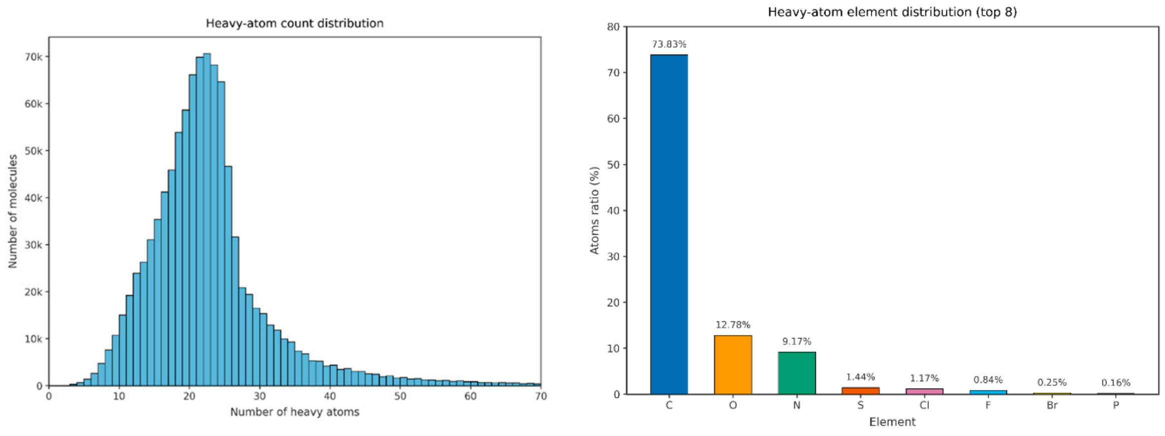


(**a**) (**b**)

**Figure S2. Statistical distribution of molecular sizes and elemental compositions in the pre-training dataset.** (a) Distribution of heavy-atom counts per molecule across the pre-training corpus. (b) Percentage distribution of the top 8 most frequent heavy-atom elements in the pre-training vocabulary.
